# A microbubble-sparged yeast propagation–fermentation process for bioethanol production

**DOI:** 10.1186/s13068-020-01745-5

**Published:** 2020-06-08

**Authors:** Vijayendran Raghavendran, Joseph P. Webb, Michaël L. Cartron, Vicki Springthorpe, Tony R. Larson, Michael Hines, Hamza Mohammed, William B. Zimmerman, Robert K. Poole, Jeffrey Green

**Affiliations:** 1 Department of Molecular Biology & Biotechnology, University of Sheffield, Sheffield, S10 2TN UK; 2 Department of Biology, University of York, York, YO10 5DD UK; 3 Perlemax Ltd, Kroto Innovation Centre, 318 Broad Lane, Sheffield, S3 7HQ UK; 4 Department of Chemical & Biological Engineering, University of Sheffield, Sheffield, S1 3JD UK

**Keywords:** Bioethanol, Conventional bubbles, Ergosterol, Fed-batch fermentation, Microbubbles, Oxygen

## Abstract

**Background:**

Industrial biotechnology will play an increasing role in creating a more sustainable global economy. For conventional aerobic bioprocesses supplying O_2_ can account for 15% of total production costs. Microbubbles (MBs) are micron-sized bubbles that are widely used in industry and medical imaging. Using a fluidic oscillator to generate energy-efficient MBs has the potential to decrease the costs associated with aeration. However, little is understood about the effect of MBs on microbial physiology. To address this gap, a laboratory-scale MB-based *Saccharomyces cerevisiae* Ethanol Red propagation–fermentation bioethanol process was developed and analysed.

**Results:**

Aeration with MBs increased O_2_ transfer to the propagation cultures. Titres and yields of bioethanol in subsequent anaerobic fermentations were comparable for MB-propagated and conventional, regular bubble (RB)-propagated yeast. However, transcript profiling showed significant changes in gene expression in the MB-propagated yeast compared to those propagated using RB. These changes included up-regulation of genes required for ergosterol biosynthesis. Ergosterol contributes to ethanol tolerance, and so the performance of MB-propagated yeast in fed-batch fermentations sparged with 1% O_2_ as either RBs or MBs were tested. The MB-sparged yeast retained higher levels of ergosteryl esters during the fermentation phase, but this did not result in enhanced viability or ethanol production compared to ungassed or RB-sparged fermentations.

**Conclusions:**

The performance of yeast propagated using energy-efficient MB technology in bioethanol fermentations is comparable to that of those propagated conventionally. This should underpin the future development of MB-based commercial yeast propagation.

## Background

In typical industrial corn/wheat mash bioethanol fermentations, yeast is propagated under aerobic conditions for 6–10 h, the yeast suspension is then diluted ~ 1:10 with fresh mash suspension, the air supply is withdrawn, and the fermentation continued for ~ 48 h. Conventional yeast propagations involve aeration systems that supply oxygen (O_2_) using inductors and spargers in an energy intensive process that can account for up to ~ 15% of total manufacturing costs [1, 2]. As the biomass increases, demand for O_2_ often outstrips the supply capacity of these systems. Increasing the surface area/volume ratio of the air bubbles introduced into fermenters increases the O_2_ transfer rate to support biomass propagation. Hence, several devices to aerate microbial cultures using microbubbles (MBs) have been developed. For example, an MB device was used to enhance O_2_ transfer and double polyhydroxybutyrate production by engineered *Escherichia coli* [3]. Production of recombinant human serum albumin by high cell density *Pichia pastoris* cultures was increased by up to sevenfold by MB aeration [4]. Microbubble sparging has also proved beneficial in xanthan gum production by *Xanthomonas campestris* [5]. Furthermore, a spinning disc MB device was shown to be able to provide cultures of *Saccharomyces cerevisiae* (up to 50 L volume) with adequate O_2_ at low agitation speed, with consequent savings in energy costs [6]. Some of these savings arise because MBs provide better mixing than regular bubbles (RB), thereby reducing local concentration gradients that could lead to O_2_-starved zones in large propagators [7].

MBs produced by fluidic oscillators with no moving parts have the potential to decrease the energetic costs of culture aeration still further [8]. A pilot study using such a system at a wastewater facility suggested that a ~ 20% decrease in blower energy costs could be achieved even under sub-optimal conditions (M. Hines, Perlemax Internal Report, 2018).

Sterol lipids contribute to resisting the toxic effects of ethanol and other stresses by maintaining the membrane rigidity [9, 10]. The biosynthesis of sterols requires O_2_ and hence this is not possible during the anaerobic ethanol-producing fermentation phase [11, 12]. Therefore, during aerobic propagation the yeast cells must synthesise sufficient sterols to provide the ethanol tolerance required during the fermentation phase.

Taken together the observations outlined above suggest that MBs could enhance O_2_ availability and reduce the overall energy costs during yeast propagation. Enhanced O_2_ supply could result in greater sterol content and thereby increase ethanol tolerance during the anaerobic production phase. However, little was known about the effects, beneficial or otherwise, of MBs on yeast biology during propagation and fermentation. Therefore, an optimised laboratory-scale RB-based propagation–fermentation process was compared with a prototype MB-based process.

## Results

### Construction of a microbubble (MB) fermenter

**Fig. 1:**
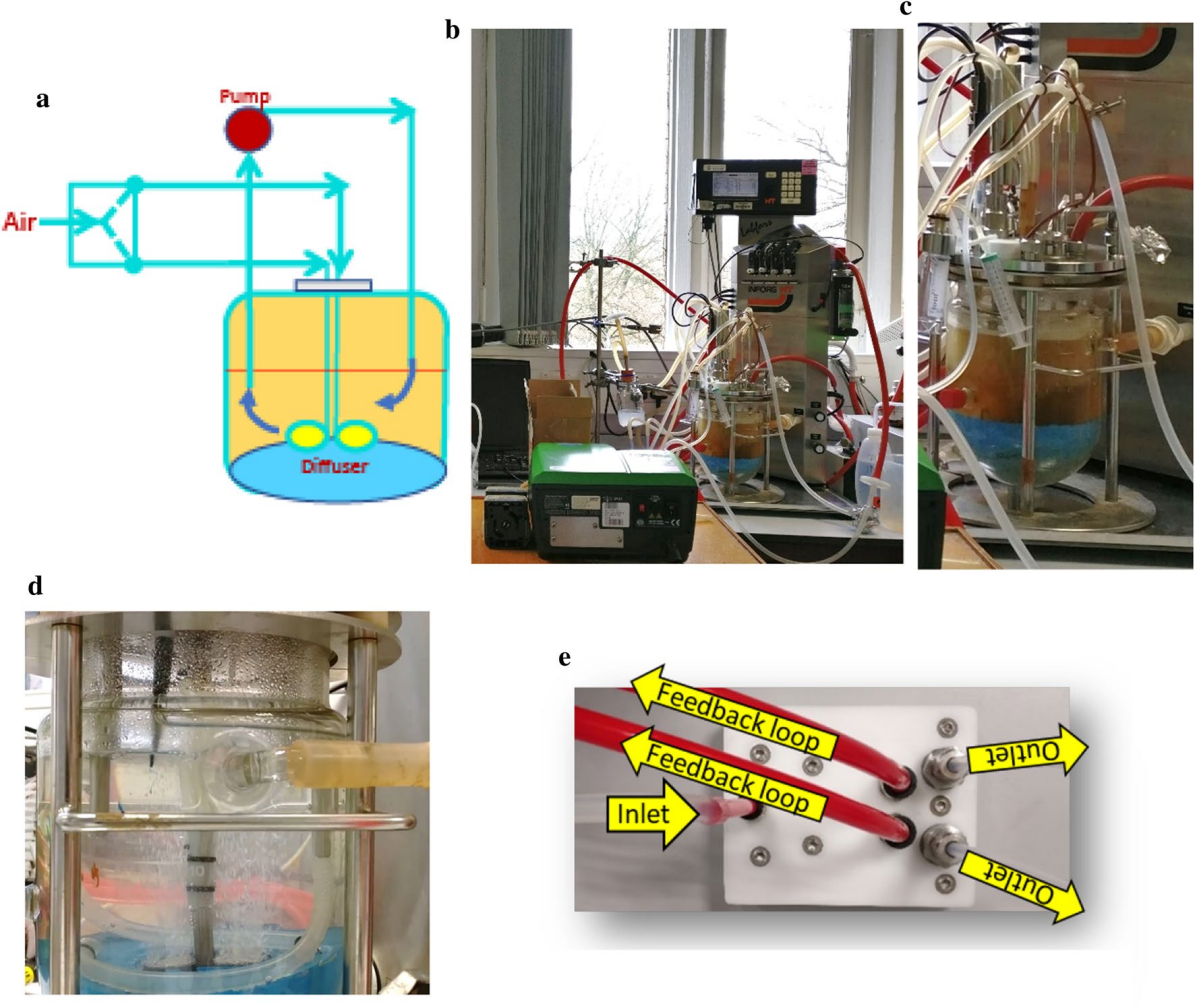
A prototype microbubble bioreactor for yeast propagation and fermentation. **a** Schematic representation of the MB bioreactor. The inlet of the fluidic oscillator is constructed to have a decreasing diameter until it reaches the junction with the two outlet tubes, which increase in diameter and are attached to the MB diffusers at the base of the vessel. At the junction, gas (air) entering the fluidic oscillator interacts with one wall and is forced along one of the outlets to emerge from the corresponding MB diffuser. A feedback loop switches the gas flow between the two outlets. A pump (red circle) recirculates culture medium from the base of the fermenter. Images showing **b** the modified Infors HT fermenter fitted with a recirculation pump; **c** the moulding (blue) fitted to the concave base of the fermentation vessel to eliminate the dead space and house the MB diffusers; **d** the sintered stainless steel diffusers and the recirculation tubing; **e** the fluidic oscillator showing the inlet connected to the gas flow meter on the bioreactor, and two outlets which send a stream of oscillating air, at a defined frequency determined by geometric features of the oscillator and the length of the feedback loop, to prevent the coalescence of bubbles as they emerge from the diffusers

The prototype MB fermenter was constructed by removing the stirrer shaft and sparger from a conventional system (Fig. 1). A plastic dome was moulded to level the concave vessel bottom and house two centrally located sintered stainless-steel diffusers. The latter were connected to the outlets of the external fluidic oscillator. A recirculation system was implemented to maintain culture homogeneity (Fig. 1). Extensive modification and testing of fluidic oscillator frequency were made before arriving at the settings used in this study [13].

### Mass transfer is enhanced in the MB fermenter

**Fig. 2:**
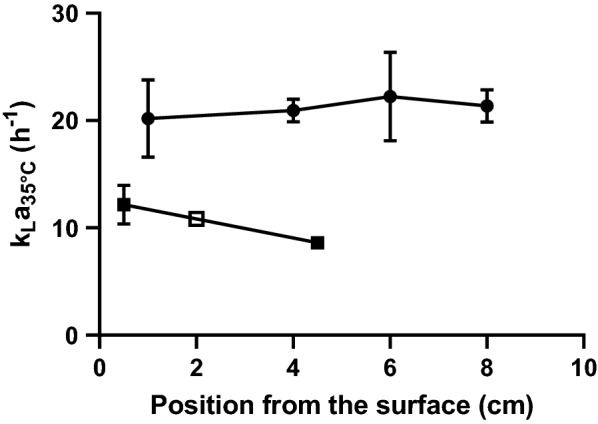
Mass transfer performance of the control and microbubble fermenters. Mass transfer (*k*_*L*_*a*) was determined using a dissolved O_2_ probe located at several positions of the fermenters at 35 °C in YPD medium. The data are the means and standard deviation (*n* = 3 for RB; for MB, *n* = 7 for the depths 1 and 6 cm, and *n* = 3 for 4 cm and *n* = 2 for 8 cm depth.). Open square represents the value that was calculated through interpolation (see text)

Mass transfer characteristics of the RB and MB-adapted fermenters were measured using a dissolved O_2_ probe located at different depths in the vessels; the position and motion of the impeller limited the analysis to two depths for the RB fermenter, whereas measurements were taken at four positions in the MB fermenter (Fig. 2). Higher *k*_*L*_*a* (the overall mass transfer coefficient) values were obtained for the MB fermenter. Furthermore, *k*_*L*_*a* remained consistent regardless of the position of the dissolved O_2_ probe for the MB fermenter, but decreased by ~ 40% at the lowest point of measurement for the RB fermenter, suggesting better mixing was achieved in the MB fermenter.

### Aerobic propagation of yeast in an MB fermenter

**Fig. 3:**
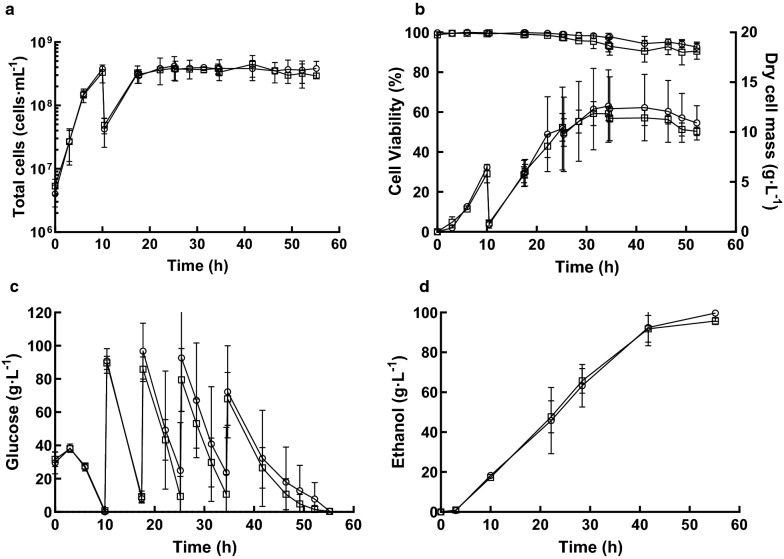
Fed-batch propagation–fermentation of *S. cerevisiae* Ethanol Red. Cultures were grown aerobically for 10 h using RB (circles) or MB (squares), followed by 45 h of ungassed anaerobic fermentation. **a** Cell density; **b** cell mass and viability; **c** residual glucose; and **d** ethanol produced. The data are the means and standard deviations (*n* = 4). Samples for transcriptome analysis were removed at 3 and 10 h during propagation and 7 and 32 h after commencing fermentation

Quadruplicate cultures of *S. cerevisiae* Ethanol Red were propagated in YPD medium containing glucose (40 g L^−1^) at 32 °C in either a RB or MB fermenter (Fig. 1). For these experiments YPD medium was used, rather than the common industrial feedstocks of cereal starches or molasses, because the composition of the latter substrates can be variable and hence introduce unknown factors that could confound identification of MB-specific effects on yeast propagation and bioethanol fermentation. For both fermenter configurations, exponential growth began immediately with a maximum specific growth rate of ~ 0.23 h^−1^ (RB: 0.24 ± 0.04 h^−1^; MB: 0.23 ± 0.05 h^−1^) producing 380 ± 36 × 10^6^ cells mL^−1^ (RB) and 332 ± 100 × 10^6^ cells mL^−1^ (MB) after 10-h propagation (Fig. 3a). Observation of the yeast by light microscopy did not show any gross morphological differences between the RB- and MB-propagated cells. For both, cell viability was ~ 100% throughout, although the budding index peaked (~ 50%) at 6 h and then decreased to ~ 40% upon glucose depletion and entry into stationary phase (Additional file 1: Figure S1). Free amino nitrogen was above 750 mg L^−1^ at the end of both propagation processes (Additional file 1: Figure S2). Cell dry masses per gram of glucose consumed (RB: 0.15 ± 0.03 g g^−1^, and MB: 0.13 ± 0.03 g g^−1^) were typical of oxidoreductive metabolism (Fig. 3b). These values reflected those of the cell counts (see above) and thus the biomass produced by MB propagation was marginally lower than that achieved by RB propagation; a similar decrease in biomass has been previously reported (RB 0.53 g g^−1^; MB: 0.43 g g^−1^ [6]), suggesting that the enhanced O_2_ transfer resulted in increased toxic reactive oxygen species. Nevertheless, it was concluded that the prototype MB fermentation apparatus could be used to propagate *S. cerevisiae* Ethanol Red with yields comparable to those of an optimised conventional RB fermenter.

### Microbubble-propagated yeast can be used for anaerobic bioethanol fermentations

To simulate industrial bioethanol fermentations, 90% of the culture was removed from the propagation vessels and replaced with fresh YPD medium containing glucose (80 g L^−1^) and gas sparging was ceased. When glucose concentrations fell below 1%, a concentrated solution of glucose was added to continue the fermentation (Fig. 3c). For both RB- and MB-propagated yeast two phases of fermentative growth were observed; a fast phase between 10 and 32 h (*µ*_max,RB_: 0.23 ± 0.04 h^−1^; *µ*_max,MB_: 0.26 ± 0.04 h^−1^), during which ethanol was produced together with cell growth, and a slower phase from 32 h until the end of the fermentation (*µ*_max,RB_: 0.03 ± 0.02 h^−1^; *µ*_max,MB_: 0.02 ± 0.01 h^−1^) where growth was uncoupled from ethanol production (Fig. 3a). The budding index remained consistent throughout at ~ 40% (Additional file 1: Figure S3). Cell viability remained high at ~ 99% in the first phase and decreased to ~ 90% at the end of the fermentation (RB: 91 ± 1%; MB: 88 ± 5%) (Fig. 3b). Cell dry mass increased from the start of the fermentation, reaching a maximum of 12.9 ± 3.4 g L^−1^ (RB) and 12.2 ± 0.7 g L^−1^ (MB) and then decreased as ethanol accumulated, possibly due to cell lysis and leakage of intracellular metabolites (Fig. 3b; Table 1). Volumetric glucose consumption rate was the highest between 10 and 17 h (RB: 11.8 ± 0.9 g L^−1^ h^−1^; MB: 11.5 ± 0.5 g L^−1^ h^−1^) and it decreased thereafter (Fig. 3c). The highest ethanol concentration achieved was 100 ± 5 g L^−1^ (RB) and 96 ± 2 g L^−1^ (MB), with a productivity of 2.2 g L^−1^ h^−1^ (Fig. 3d; Table 1). Thus, it was concluded that the performance of MB-propagated yeast in anaerobic bioethanol production was comparable to that of RB-propagated cells.

**Table 1:** Physiological parameters during fed-batch fermentation with *S. cerevisiae* Ethanol Red

Propagation gassing type	Fermentation gassing type	Maximum cell number (10^6^ cells mL^−1^)	Dry cell biomass (g L^−1^)	Ethanol (g L^−1^)	Viability (%)
21% O_2_ RB	Ungassed	492 ± 141	12.9 ± 3.4	100 ± 5	91 ± 1%
21% O_2_ MB	Ungassed	498 ± 135	12.2 ± 0.7	96 ± 2	88 ± 5%
21% O_2_ MB	1% O_2_ RB	641 ± 77	14.4 ± 2.3	96 ± 3	78 ± 4
21% O_2_ MB	1% O_2_ MB	721 ± 85	14.8 ± 1.3	89 ± 3	75 ± 7

### Enhanced expression of ergosterol biosynthesis genes in MB-propagated yeast

The macro-physiological parameters indicated that the MB propagation–fermentation process was as effective as a conventional process in an RB reactor. To determine whether these similar macroscopic outputs required transcriptional reprogramming in response to the different physical properties of MBs compared to RBs, global gene expression profiles were obtained for early and late propagation, and early and late fermentation cells (Table 2). Comparing gene expression of the early (*t* = 3 h) MB-propagated yeast to that of RB-propagated yeast indicated that 15 genes were differentially regulated (≥ twofold, adjusted *p *≤ 0.05; Additional file 1: Table S1), whereas 104 genes were differentially regulated in late propagation (*t* = 10 h; Additional file 1: Table S2). Gene ontology analysis revealed enrichment in metal ion homeostasis [GO: 0055072] during early propagation (Additional file 1: Table S3), whilst cellular amino acid biosynthesis [GO: 0008652] and ergosterol biosynthesis [GO: 0006696] were enriched during late propagation (Additional file 1: Table S4). Thus, although the macro-physiology of the cells was unaffected by the mode of aeration, MB aeration elicited significant changes in gene expression during the propagation phase, including enhanced expression of genes required for ergosterol synthesis.

**Table 2:** Differentially expressed genes during aerobic batch propagation and fed-batch fermentation (ungassed) with *S. cerevisiae* Ethanol Red using YPD medium (≥ twofold adjusted *p* ≤ 0.05)

Sample	Number of genes up-regulated	Number of genes down-regulated	Total genes
Early propagation (*t* = 3 h)	2	9	11
Late propagation (*t* = 10 h)	48	56	104
Early fermentation (*t* = 7 h)	21	13	34
Late fermentation (*t* = 32 h)	1	–	1

Gene expression of the MB-propagated yeast was then compared to that of RB-propagated cells in the anaerobic fermentation phase. During early fermentation (*t* = 7 h), 34 genes were differentially expressed in yeast that had been MB-propagated compared to RB-propagated (Additional file 1: Table S5). GO analysis revealed that plasma membrane organisation [GO:0007009] and responses to stress [GO: 0006950] were enriched (Additional file 1: Table S6). In the late fermentation phase (*t* = 32 h), only *CYB2* (Additional file 1: Table S7), a component of the mitochondrial intermembrane space, was significantly different. The expression of genes associated with pyruvate fermentation (*PDC1, 5, 6*; *ALD4*, 5; *ADH1, 2, 3, 4, 5*; *BDH1*) was mostly unchanged after transition from late propagation to early fermentation, but both RB- and MB-propagated cells exhibited two to threefold increased expression of *PDC5* (pyruvate decarboxylase) and *ADH1* (alcohol dehydrogenase), whose actions combine to convert pyruvate to ethanol (Additional file 2).

### Enhanced abundance of ergosteryl esters in MB fermentations

The higher level of expression of ergosterol biosynthesis genes in MB-propagated yeast suggested that such yeast could possess a larger reservoir of sterols and therefore exhibit enhanced ethanol tolerance during anaerobic fermentation. Yeast membranes exposed to ethanol exhibit increased lipid head group spacing, membrane fluidity and permeability, eventually leading to the lipid bilayers becoming interdigitated. Together these effects impair membrane function and yeast viability limiting the yields of bioethanol fermentations [14]. Ergosterol counteracts ethanol-induced interdigitation of lipid bilayers and enhanced levels of *S. cerevisiae* ergosterol correlated with increased ethanol tolerance [9]. However, the physiological and gene expression data indicated that RB- and MB-propagated yeast performed similarly in anaerobic fermentations.

**Fig. 4:**
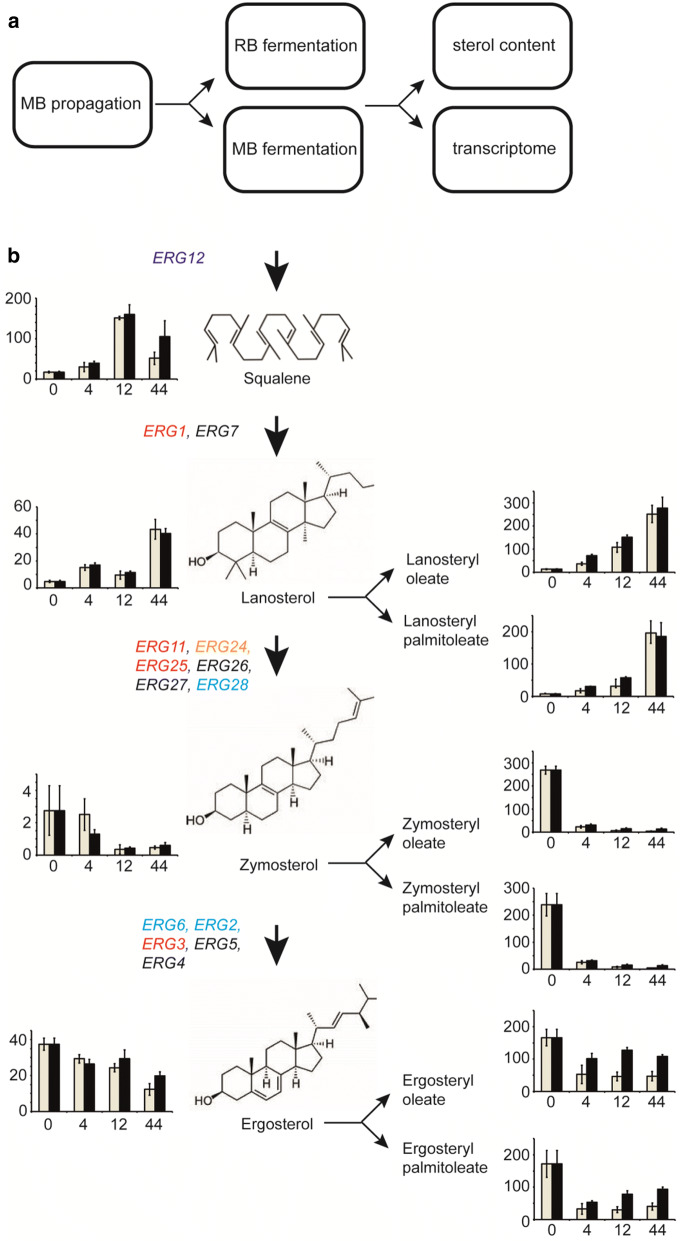
Effect of gassing with 1% O_2_ regular bubbles (RB) or microbubbles (MB) on yeast sterol content during ethanol-producing fermentations. **a** Schematic diagram showing the experimental approach. Yeast cultures were propagated aerobically in the prototype MB fermenter sparged with 21% O_2_ as described in the text. The same yeast cells were used to seed fermentation runs sparged with 1% O_2_ supplied as either RBs or MBs. Samples were removed for sterol and transcriptome analyses 0, 4, 12 and 44 h after commencing fermentation. Experiments were performed in triplicate. **b** Effects of sparging fermentations with 1% O_2_ on expression of sterol biosynthesis genes and sterol content. The bar charts show the amounts (μg mg^−1^ cell dry mass; vertical axes) of the indicated sterols and sterol esters plotted against fermentation time (h). The data are the means and standard deviations for three biological replicates (grey bars, RB fermentation; black bars, MB fermentation). Differentially expressed (≥ twofold, adjusted *p* ≤ 0.01, *n* = 3) ‘*ERG*’ genes are indicated to the left of the simplified ergosterol biosynthesis pathway: black type, no change; red type, up-regulated in both RB and MB fermentations; cyan type, up-regulated in MB fermentation only; orange type, up-regulated in RB fermentation only

Synthesis of sterols requires O_2_. Therefore, the effect of ergosterol biosynthesis gene expression on enhanced ethanol production when O_2_ is supplied by RBs or MBs during the fermentation phase was investigated. MB-propagated yeast was used as the inocula for fermentations gassed with 1% O_2_ supplied by RBs or MBs (Fig. 4a). The amount of O_2_ supplied was theoretically sufficient for the biosynthesis of ergosterol and oleate [12]. Sterol contents were analysed at early (*t* = 0, 4 h), mid (*t* = 12 h), and late fermentations (*t* = 44 h). Similar patterns of squalene, lanosterol, lanosteryl palmitoleate, lanosteryl oleate, zymosterol, zymosteryl oleate, zymosteryl palmitoleate and ergosterol content were observed for RB- and MB-sparged fermentations (Fig. 4b). Zymosterol and its esters became less abundant as the fermentations progressed, whereas squalene, lanosterol and their esters increased in abundance. In both fermentation processes, ergosterol amounts were maintained during the first 12 h of fermentation but, after an initial decrease, higher levels of ergosteryl palmitoleate and ergosteryl oleate were present when O_2_ was supplied by MBs (Fig. 4b). As free ergosterol is cytotoxic, esterified sterols possibly act as reserves during MB-gassed fermentations [14].

### Changes in gene expression during oxygen-gassed fermentations

**Fig. 5:**
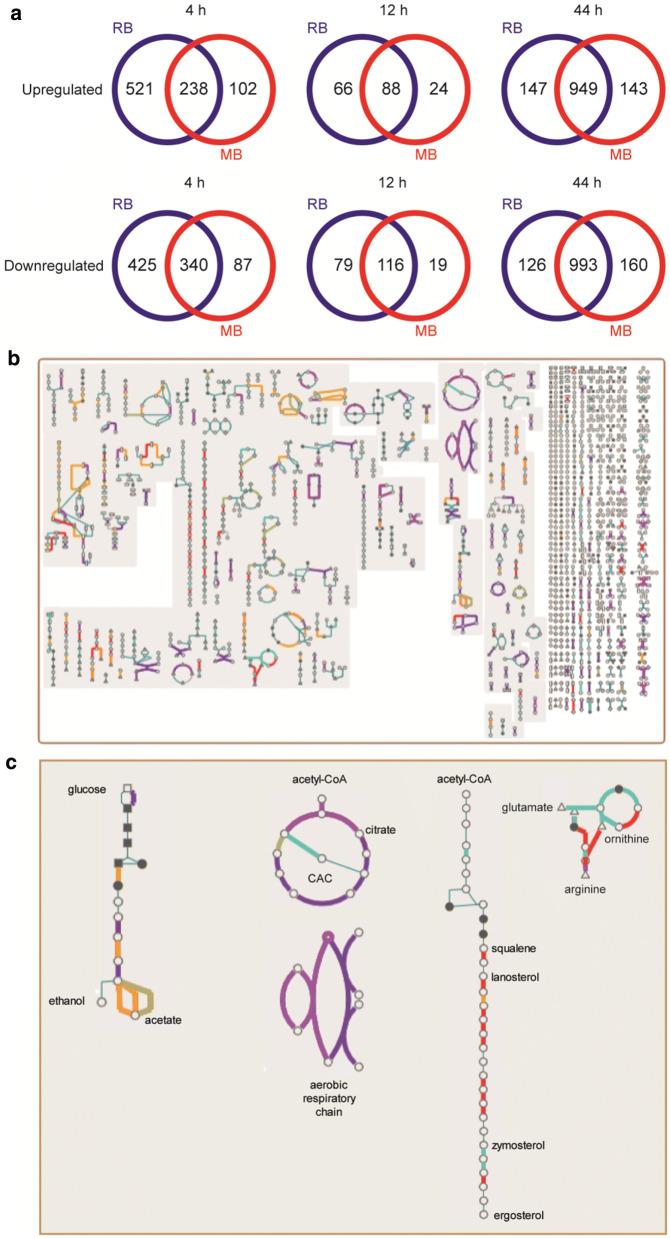
Changes in gene expression during gassed fermentation of MB-propagated yeast. An overview of the experimental approach is provided in Fig. 4a. **a** Venn diagrams showing differential gene expression (≥ twofold, adjusted *p* ≤ 0.01) at the indicated times for fermentations gassed with 1% O_2_ using RB (blue) or MB (red). **b** Cellular pathway overview of *S. cerevisiae* metabolism from yeast pathways [17] showing reactions associated with differentially genes 4 h into the fermentations compared to the MB-propagated inocula: up-regulated in both RB and MB fermentations (red); down-regulated in both RB and MB fermentations (dark blue); up-regulated in RB (orange) or MB (cyan) fermentations only; down-regulated in RB (purple) or MB (green) fermentations only. **c** Higher resolution representations of (left to right) glucose fermentation, citric acid cycle (CAC) and aerobic respiratory chain, sterol biosynthesis and arginine metabolism. Colour key as stated in **b**

Gene expression profiles during the fermentations gassed with 1% O_2_ supplied by RBs or MBs were compared. Widespread changes in gene expression (≥ twofold; adjusted *p *≤ 0.01) were observed in response to the lower O_2_ supply, i.e. shift from aerobic propagation (21% O_2_) to sparged fermentation (1% O_2_) (Fig. 5a). However, initially the changes were fewer for the MB-sparged fermentations, likely due to the more efficient gas transfer compared to RBs. At the end of the fermentations > 2000 genes were differentially expressed (≥ twofold; adjusted *p* ≤ 0.05) compared to the aerobic inocula (Additional file 3).

During early fermentation (*t* = 4 h), 690 genes were significantly (≥ twofold; adjusted *p *≤ 0.05) regulated (Additional file 3) involved in a wide range of cellular processes (e.g. response to stress GO:0006950, protein refolding GO:0042026, ribosome biogenesis GO:0042254, mitochondrial electron transport GO:0006122; Additional file 1: Table S8). During mid-fermentation (*t* = 12 h), 24 genes were differentially expressed with an enrichment in heme (GO:0042167, GO:0006788) and sterol metabolism (GO:0016126) (Additional file 1: Table S9). At the end of fermentation, 53 genes were differentially expressed relating to processes involved in DNA damage and disaccharide metabolism (Additional file 1: Table S10). Ranking differentially expressed genes based on DNA binding and expression changes mediated by *S. cerevisiae* transcription factors in Yeastract [15] showed that no regulons were significantly enriched early (4 h) or late (44 h) into the gassed fermentations. However, the Hap1p regulon was differentially regulated in the mid-fermentation (12 h) samples (Additional file 1: Table S11). Hap1p is a zinc-finger transcription factor that is essential for anaerobic growth and activates the expression of aerobic respiratory proteins by indirectly sensing O_2_ availability through the capacity to synthesise heme [16]. The higher expression of *CYB2* (3.8-fold), *CYC1* (3.1-fold), *COX26* (4.8-fold) and *HMX1* (4.8-fold), and lower expression of *AAC3* (2.8-fold) in the MB-sparged fermentations, compared to the RB-sparged cultures, suggest that sufficient O_2_ supply is maintained for longer in the MB fermenter as a consequence of the superior mass transfer values associated with MBs (Fig. 2).

As noted above, 4 h into the MB-gassed fermentation the ergosterol biosynthesis genes, *ERG* 2, 6, 12 and 28 were up-regulated, *ERG24* was up-regulated in the RB fermentations and *ERG* 1, 3, 11 and 25 exhibited enhanced expression in both fermentations (Figs. 4b and 5c). Expression of *YEH1* (steryl ester hydrolase) increased in both processes, suggesting that there was greater recycling of steryl esters. Nevertheless, *ARE2* (acylCoA:sterol acyltransferase), which catalyses the synthesis of steryl esters, was significantly up-regulated in the MB fermentations, and could account for the higher amounts of ergosteryl palmitoleate and ergosteryl oleate in these fermentations (Fig. 4b). At the end of both fermentations *ERG 1, 2, 4, 5, 6, 8, 9, 10, 11, 12 13, 20, 26, 27* and *28* were down-regulated.

Mapping of significantly regulated genes (≥ twofold, adjusted *p* ≤ 0.01) 4 h into the fermentations to the cellular overview of *S. cerevisiae* metabolism available in Yeast Pathways (https://pathway.yeastgenome.org/ [17]) showed that the common responses to the switch from 21% O_2_ sparging to 1% O_2_ included down-regulation of citric acid cycle and aerobic respiratory genes and down-regulation of trehalose biosynthesis genes (Fig. 5b). Whilst up-regulation of several genes involved in sterol and arginine biosynthesis was common to both fermentation processes, more of these genes were up-regulated in the MB fermentations (Fig. 5c). One of the most up-regulated genes in both fermentations was *HES1* (*OSH5*), coding for a protein that resembles the mammalian oxysterol binding protein (OSBP) which is implicated in ergosterol homeostasis, with an *HES1* (*OSH5*) mutant exhibiting lower ergosterol content, but similar lanosterol and zymosterol contents, to wild-type *S. cerevisiae* [18]. Increased amounts of sterols and arginine have been reported to enhance ethanol tolerance and hence the increased expression of these genes in the MB fermentations could be a useful trait conferred by an MB propagation–fermentation [9, 19].

**Fig. 6:**
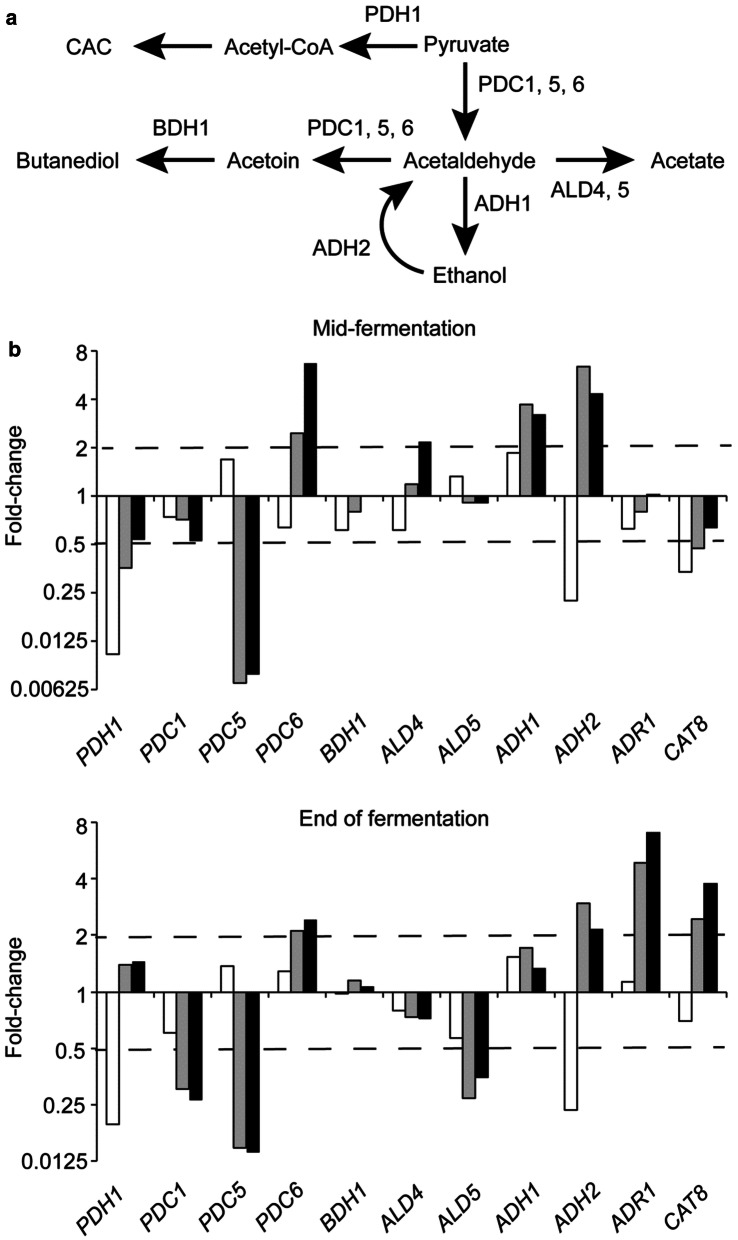
Oxygen sparging during fermentation enhances expression of *PDC6* and *ADH2*, genes. **a** Simplified diagram of pyruvate metabolism and relevant enzymes: pyruvate dehydrogenase, PDH1; pyruvate decarboxylase, PDC; aldehyde dehydrogenase, ALD; alcohol dehydrogenase, ADH; butanediol dehydrogenase, BDH1; citric acid cycle, CAC. **b** Changes in expression of the indicated genes (fold change relative to the MB-propagated inoculum) at mid- and end of ungassed (open bars), RB (1% O_2_; grey bars) and MB (1% O_2_; black bars) fermentations. The dashed lines mark ≥ twofold up- or down-regulation

Changes in the expression of genes linked to pyruvate metabolism upon transition to 1% O_2_ sparging were similar for both RB and MB fermentations, but differed from the anaerobic fermentations (Fig. 6). The pyruvate dehydrogenase gene (*PDH1*) was more severely repressed in the ungassed fermentations, potentially increasing flux to ethanol. Furthermore, the pyruvate decarboxylase genes *PDC5* and *PDC6* showed opposite regulation when MB-propagated cells were used in anaerobic fermentations (*PDC5* up-regulated, *PDC6* down-regulated) compared to the 1% O_2_-sparged fermentations (*PDC5* down-regulated, *PDC6* up-regulated). Expression of *PDC6* is usually lower than *PDC1* and *PDC5*, which are considered to be more important for ethanol production by catalysing the conversion of pyruvate to acetaldehyde (Fig. 6a; [20]), whereas *PDC6* supported the growth of a *PDC1/PDC5* mutant on ethanol medium [21]. The alcohol dehydrogenase gene *ADH1* was up-regulated in all the fermentations, but in the gassed fermentations expression of *ADH2* increased, whereas it decreased in the ungassed fermentation (Fig. 6b). Adh1p is responsible for conversion of acetaldehyde to ethanol, whereas the kinetic properties of Adh2p are thought to favour the reverse reaction permitting aerobic utilisation of ethanol [22]. Transcription of *ADH2* is co-regulated by Adr1p and Cat8p in response to glucose depletion [23]. Expression of *ADR1* and *CAT8* was enhanced at the end points of the gassed fermentations, but was unchanged in the ungassed fermentation (Fig. 6b). The expression patterns of *PDH1*, *PDC6* and *ADH2* suggest that, whilst the gassing regime employed here enhanced the content of ergosterol esters, it also facilitated the consumption of ethanol and aerobic metabolism.

### Oxygen sparging during fermentation decreased yeast viability

**Fig. 7:**
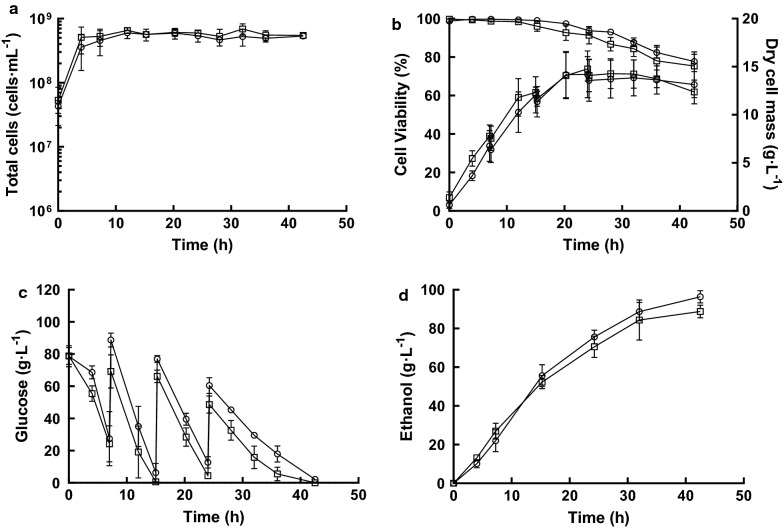
Fed-batch fermentations of *Saccharomyces cerevisiae* sparged with low levels of O_2_. The yeast was MB-propagated for 10 h and then used to inoculate fermentations sparged with either RBs (circles) or MBs (squares) consisting of a 1% O_2_, 99% nitrogen gas mix. **a** Cell density; **b** dry cell mass and viability; **c** residual glucose; and **d** ethanol produced. Only the micro-aerobic phase is shown. The data are the means and standard deviations (*n* = 3)

Microbubble-propagated cells exhibited increased expression of ergosterol biosynthetic genes compared to RB-propagated cells, but this did not result in enhanced ethanol production in a typical anaerobic fermentation (Fig. 3). Moreover, introducing low levels of O_2_ using MBs during fermentation enhanced expression of a subset of genes required for sterol ester synthesis and increased the content of ergosteryl palmitoleate and ergosteryl oleate of the yeast cells compared to RB cultures (Fig. 4b). However, the enhanced expression of *PDC6* and *ADH2* suggested that continuous sparging with 1% O_2_ during fermentation allowed the metabolism of ethanol (Fig. 6b). Previous studies have used various aeration regimens to improve ethanol production [24–27]; however, relatively little is known of the effects of O_2_ on yeast exposed to high ethanol concentrations. Therefore, fermentations sparged with RBs and MBs consisting of 1% O_2_–99% N_2_ were analysed for ethanol production and yeast viability. Just as in the ungassed fermentations, two growth phases were observed. A fast growth phase, in which cells produced ethanol together with higher biomass (14.4 ± 2.3 g L^−1^ [RB] and 14.8 ± 1.3 g L^−1^ [MB]) compared to ungassed fermentations (Fig. 7). The final cell densities were also higher than those obtained for non-oxygenated fermentations (641 ± 77 × 10^6^ cells mL^−1^ [RB] and 721 ± 85 × 10^6^ cells mL^−1^ [MB] (Fig. 7a)), indicating that metabolism was respiro-fermentative. The maximum ethanol concentrations were 96 ± 3 g L^−1^ (RB) and 89 ± 3 g L^−1^ (MB) (Fig. 7d), which were slightly lower than those of the ungassed fermentations (Fig. 3d). The lower concentration of ethanol measured in the MB fermentations was at least in part caused by ethanol stripping. Indeed, ethanol concentrations from RB-gassed cultures plotted against those from MB-gassed cultures deviated 5% from the identity line (*y* = *x*), whilst for the ungassed fermentation, the deviation was less than 0.5% (Additional file 1: Figure S4). Unexpectedly, cell viability for the O_2_-gassed fermentations cell viability decreased more rapidly (~ 1% h^−1^) compared to ungassed (< 0.3% h^−1^) or O_2_-free N_2_-gassed (< 0.2% h^−1^) cultures and hence the loss of viability was attributable to the presence of O_2_ (Fig. 7b; Additional file 1: Figure S5). It is known that reactive oxygen species are generated during ethanol production [28] and that these damage a wide range of cell components; it is likely that reactive oxygen species production and the resulting cell damage are exacerbated due to limited oxygenation during the fermentation phase [29].

## Discussion

*Saccharomyces cerevisiae* Ethanol Red is used for commercial production of bioethanol. The process has two stages; the yeast is cultured in aerated vessels and these are subsequently used to seed anaerobic fermentations during which feedstock sugars are converted to ethanol. A significant manufacturing cost is the provision of air (O_2_) during propagation [1, 2]. Advances in MB technology offer opportunities to reduce these costs and thereby improve the economics of bioethanol production [7]. However, a molecular physiological analysis of MB-propagated yeast in a fed-batch bioethanol process had not been undertaken previously. The data reported here show that a prototype system fitted with an energy-efficient fluidic oscillator supported enhanced O_2_ transfer to the yeast culture and that the resulting biomass performed comparably to conventionally propagated yeast in anaerobic fermentations. Under industrial conditions, in which propagation–fermentation is supported by variable, poorly defined, corn- or wheat-based mash, the superior mass transfer achieved using MBs could be advantageous in maximising biomass yields. Preliminary laboratory propagation trials using wheat mash in a vessel fitted with a fluidic oscillator and diffuser suggested a marked improvement in mass transfer and cell numbers compared to conventional propagation (unpublished data). Therefore, the performance of the prototype laboratory-scale system described here demonstrates the potential utility of fluidic oscillator generated MBs and their associated cost benefits for application in bioethanol production.

The comparable macro-physiological characteristics of the MB-propagated yeast were accompanied by differences in membrane composition that could provide a platform for further process development. Sterols are membrane lipids whose synthesis requires O_2_ and contribute to resisting the toxic effects of ethanol [9, 10]. The MB-propagated yeast exhibited enhanced expression of ergosterol biosynthesis genes and possessed increased amounts of ergosteryl esters compared to those propagated conventionally. However, these enhanced pools of sterol esters did not translate into increased ethanol production in the anaerobic fermentations reported here and nor did attempt to exploit the enhanced expression of sterol biosynthesis genes by introducing 1% O_2_ MBs during the production phase. Nevertheless, these observations suggest that with further process development to counteract the detrimental effects of reactive oxygen species and ethanol consumption in O_2_-sparged fermentations, MB-propagated yeast might exhibit improved ethanol tolerance. Such developments might include optimising the rate and timing of the O_2_ supply to MB-propagated yeast during the fermentation phase, as these factors have previously shown to important for biomass and ethanol production in very-high-gravity ethanol fermentations [26]. Hence, the work described here should inform the next stage in MB reactor design and process development by providing the reassurance that MB-propagated yeast perform at least as well as those grown conventionally.

## Conclusion

Application of a microbubble (MB) aeration system with no moving parts enhanced O_2_ transfer to cultures of *S. cerevisiae* Ethanol Red. The MB-propagated yeast performed similarly to yeast propagated conventionally when used as the seed culture for bioethanol fermentations. This study provides the biological underpinning for future development of energy efficient, higher yielding commercial-scale MB-based yeast propagation.

## Methods

### Microorganisms and maintenance

*Saccharomyces cerevisiae* Ethanol Red was obtained from Ensus UK. Strains were stored as glycerol (30% v/v) stocks (− 80 °C). Strains were routinely grown on YPD [yeast extract 10 g L^−1^, peptone 20 g L^−1^ and Sigma glucose (dextrose) 20 g L^−1^]. When solid medium was required agar (20 g L^−1^) was added. Routine growth was performed at 30 °C, 200 rpm.

### Inoculum preparation

Cells from a single colony were inoculated into YPD (10 mL) and grown for 17 h. Cells were counted in a Neubauer chamber using a phase contrast microscope at 400× magnification. The required volume of culture liquid—corresponding to an initial pitching density of 5 × 10^6^ cells mL^−1^ at the start of the propagation—was centrifuged, pellet resuspended in sterile YPD (1 mL) and used as the inoculum for batch fermentations.

### Mass transfer determination

Mass transfer was determined in triplicate for a variety of flow rates and diffuser configurations at 35 °C in 40 g L^−1^ glucose supplemented YPD, as per the propagation and fermentation experiments. For each configuration, the media were allowed to stably come to temperature before proceeding. Using an optical dissolved O_2_ (DO) probe (PreSens, Germany), the DO was able to be measured in various positions. The position of the probe in the control system was limited to two points (0.5 and 4.5 cm from the liquid surface) due to the movement and location of the impeller. However, the position of the probe in the MB system was captured at four different vertical positions (1, 4, 6 and 8 cm from the surface). The control configuration used the standard “J” type sparge tube that comes as standard with the Infors bioreactor to deliver gas to the system. All control experiments were stirred at 400 rpm (standard Rushton type impeller). To limit biomass settling, the medium in the MB fermenter was recirculated using a peristaltic pump (58 mL min^−1^). The DO in the medium was lowered to 0 ± 0.05 mg L^−1^ using pure nitrogen. Using the Infors mass flow meter, the desired flow rate of air was delivered to the system. The dissolved O_2_ was then allowed to rise to ~ 98% of saturation. Mass transfer was calculated using Eq. 1: where *C*_*t*_ is the concentration of dissolved O_2_ at time *t*, *C*_Sat *T*_ the concentration of dissolved O_2_ at saturation at temperature *T*, *C*_0_ the zero saturation dissolved O_2_ concentration, *k*_*L*_*a*_*T*_ the interfacial mass transfer at temperature *T* and *t* time. The interfacial mass transfer was determined by regression and minimisation of the residual of the sum of squares.

1 $$ C_{t} = C_{{{\text{Sat}}T}} - \left( {C_{{{\text{Sat}}T}} - C_{0} } \right)e^{{ - k_{L} a_{T} t}} $$

### Conventional propagation and fed-batch fermentation

Batch aerobic propagation was carried out in a 2-L Infors fermenter with a working volume of 1 L using YPD medium supplemented with 40 g L^−1^ of glucose. The bioreactor was sterilised by autoclaving (45 min, 121 °C). The temperature was controlled at 32 °C and the cultivation medium was sparged with filtered air (0.2 vvm). The agitation rate was maintained at 400 rpm. The culture vessel was inoculated with ~ 1 mL of culture corresponding to an initial pitching density of 5 × 10^6^ cells mL^−1^. The exhaust gas was passed through a condenser, maintained at 10 °C by circulating cooled water. Samples (5 mL) were taken for biomass, absorbance, and metabolite analysis every 3 h during the exponential growth phase. Data acquisition of process variables was recorded automatically using Iris or Eve software. After 10 h of yeast propagation, 90% of the culture was removed by creating an over pressure in the bioreactor by blocking the exhaust. The reactor was then fed with fresh YPD medium (800 mL; 1:9 dilution of the propagated yeast suspension; autoclaved for 20 min at 121 °C) containing glucose (80 g L^−1^) to commence the anaerobic fermentation phase, without gas sparging. When glucose levels reached less than 1.0% (determined using a portable refractometer), the bioreactor was pulsed with a known volume of a concentrated glucose solution (750 g L^−1^; autoclaved for 20 min at 121 °C) via a high-speed peristaltic pump. The dispensing volumes and the time of pulse additions after the start of fermentation phase were 75 mL at 7 h, 100 mL at 15 h and 75 mL at 24 h. Samples (5 mL) were taken at regular intervals to monitor the fermentation profile and for analytical measurements.

### Microbubble propagation and fed-batch fermentation

For MB batch propagations the cultivation conditions were the same as that for conventional propagations but with two major alterations: the stirrer shaft and the sparger were removed to accommodate the sintered stainless steel diffusers housed within a custom-built dead space eliminator at the bottom of the vessel. Custom-built metal plates and Teflon spacers on the head plate held the diffuser in place ensuring a hermetic seal for culture sterility. Tubes emerging from the two diffusers were connected to the two outlets of the fluidic oscillator [13]. The fluidic oscillator was sterilised by filling it with ethanol (70% v/v) and leaving it for 24 h. Ethanol was drained from the fluidic oscillator just before the start of the batch process and connected to the tubing from the diffuser. Two metal tubes from two ports on the head plate were connected in a closed loop via norprene tubing. A peristaltic pump recirculated the cell suspension from the bioreactor via the closed loop, to ensure cell homogeneity.

### Gassed fermentation

Yeast cells were propagated using for 10 h in an MB bioreactor. After 10 h, 90% of the contents were removed as described for the ungassed fermentations and used as inocula for RB- and MB-oxygenated fermentations. During the fermentations, a gas mixture containing 1% O_2_ and 99% N_2_ was sparged to supply small amount of O_2_. To promote stripping of ethanol via gassing, the condenser cooling was turned off. N_2_-gassed fermentation was carried out exactly as above but sparged with ultrapure N_2_ (BOC certified O_2_ free N5.5).

### Biomass determination

A 3-mL sample was filtered using a pre-dried, pre-weighed 0.45-µm filter membrane and washed with distilled water. The filter membrane with the wet biomass was dried in a microwave oven at 150 W for 10 min. The biomass concentration was calculated from the difference of the masses and the volume of the broth used.

### Cell viability and budding index

Viable cells exclude the dye methylene blue [30]. Diluted samples (50 µL) from the propagation–fermentation runs were mixed with 50 µL of methylene blue (0.01% (m/v)) and incubated for 5 min and the number of stained and unstained cells was counted. Budding index was scored by counting a minimum of 300 cells. Assays were performed in duplicate.

### Extracellular metabolites’ determination

Glucose was analysed using the Megazyme GOPOD kit (K-GLUC 10/15, Megazyme Inc., Ireland); ethanol was analysed using the Megazyme kit (K-ETOH, Megazyme Inc., Ireland) using the manual assay procedure for large volumes in a cuvette. The assays were performed in duplicate.

### Transcript profiling using microarrays

Two time points from the propagation phase (3 h, 10 h) and two time points from the fermentation phase (7 h, 32 h) were chosen for gene expression analysis. All analyses were performed in triplicate except the early propagation sample (3 h) for which only duplicate samples were available. There were 24 samples in total including two technical replicates. Culture samples for transcriptional profiling were directly eluted into 2 volumes of RNAprotect (Qiagen) to rapidly stabilise the mRNA. Total RNA was prepared using the RNeasy RNA purification kit (Qiagen), according to the manufacturer’s instructions (including the on-column DNAse treatment step). The eluted RNA was treated again with DNase and re-purified. Quality of RNA was checked using agarose gel electrophoresis and PCR using DNA specific primers. RNA was quantified on a NanoDrop 1000 spectrophotometer (Thermo Fisher Scientific). Labelled cDNA was produced using SuperScriptIII reverse transcriptase (Invitrogen) with the Cy3-dCTP included in the dNTP mixture. Labelled *S. cerevisiae* genomic DNA was produced using BioPrime DNA Labelling Kit (Invitrogen) with Cy5-dCTP included in the dNTP mixture. Labelled genomic DNA and cDNA were combined and hybridised overnight to an oligonucleotide microarray (Agilent Technologies). Quantification of cDNA samples, hybridisation to microarrays, microarray processing and scanning were carried out as described in the Fairplay III labelling kits (Agilent Technologies, 252009, Version 1.1) and scanned with a high-resolution microarray scanner (Agilent Technologies).

Features with background intensities exceeding 10 times the array median, or with a signal to background ratio below 3 were excluded from further analysis. Background correction [31] within-array loess normalisation [32] and between-array quantile normalisation was applied to the remaining features using the R statistical package LIMMA from Bioconductor [33]. Moderated t-statistics were calculated using gene-wise linear models with an empirical Bayes approach [34, 35].

### Transcript profiling by RNA sequencing

Samples taken from the bioreactor (1 mL) were immediately harvested by centrifugation and the pellets flash frozen in liquid nitrogen. RNA isolation, polyA selection, transcript library preparation and paired-end sequencing on an Illumina Hi-Seq were performed by GENEWIZ.

Trimmed reads were aligned to the *Saccharomyces cerevisiae* S288C reference genome (NCBI assembly: GCA_000146045.2) using TopHat2 [36]. Numbers of mapped reads aligned to each gene were counted using HTSeq [37]. Raw counts were converted to log_2_ counts per million using the LIMMA voom transformation [38], and further differential expression analysis was performed using the LIMMA package in R.

### Analysis of transcriptomic datasets

For both microarray and RNAseq analyses *p* values were adjusted for multiple testing using the Benjamini–Hochberg method [39]. Transcripts exhibiting ≥ twofold change in abundance with an adjusted *p* value < 0.05 were deemed to be differentially regulated. GO enrichment analysis was performed using the differentially expressed gene lists in Funspec [40]. Pathway enrichment analysis was performed using Metacyc [41] to identify significantly enriched metabolic or signal transduction process. Transcription factors likely to be involved in mediating the observed changes in gene expression were ranked using Yeastract [15].

### Lipidomics

Sterol analysis was performed on lipid extracts from lyophilised cell material. Samples were weighed (5 mg) into 2-mL microfuge tubes, together with 10 µL of internal standard mix containing 1 µg deuterated cholesterol and 3.5 µg deuterated cholesterol steryl ester (SPLASH lipidomix, p/n 330707; Avanti Polar Lipids, AL, USA). Water (50 µL), CHCl_3_:MeOH (2:1 v/v, 700 µL) and acid-washed glass beads (300 mg, Sigma; 425–600 µm) were added to each tube. Samples were then extracted in a bead mill (Qiagen TissueLyser II; 2 × 3 min pulses at 30 Hz with intervening plate rotation), snap-frozen in liquid N_2_, then allowed to slowly thaw at 4 °C for 24 h. Samples were subsequently centrifuged at 16,000×*g* for 10 min, the supernatant transferred into fresh 2-mL tubes, and developed into two phases following addition of 300 µL 0.9% KCl (w/v) and vortexing briefly. The lower phase was transferred into glass HPLC vials, and vacuum evaporated to dryness on a GeneVac EZ2 centrifugal evaporator at the very low boiling point setting. Samples were reconstituted in 200 µL acetonitrile:isopropanol (7:3, v/v), and 2 µL analysed by LCMS. LC separation was performed on an Accucore C30 column (Thermo Scientific; 100 mm × 2.1 mm, 2.6 µm particle size) and masses acquired in data-dependent MS2 mode on a Thermo Orbitrap Fusion Tribrid mass spectrometer as previously described [42], except an atmospheric chemical pressure ionisation (APCI) source was used to generate ions for measurement in positive mode only, and MS1 data were acquired at a mass resolution of 60,000 FWHM. Ergosterol was identified by reference to an authentic standard (Sigma), and all candidate sterols identified by homology as their [M-H_2_O + H]^+^ ions (MS1 quant ions for sterols and deuterated cholesterol). Sterol esters had diagnostic in-source fragments [Sterol-OH]^+^ (also used for MS1 quant) and [M + C_3_H_3_]^+^ adduct ions (identified in deuterated cholesterol steryl ester). Peak areas were converted to amounts using Thermo Xcalibur 4.0 QuanBrowser software, using 20 ppm mass tolerances for quant ions relative to the internal standard area responses of deuterated cholesterol for all sterols and cholesterol steryl ester for all sterol esters, respectively. Squalene was quantified from its [M + C_3_H_3_]^+^ quant ion ([M + H]^+^ and M + NH_4_]^+^ diagnostic ions were also observed), relative to deuterated cholesterol in the internal standard.

### Calculation of physiological parameters

The maximum specific growth rate was obtained by plotting the natural logarithm of the dry biomass against time. The slope of the linear regression line represents the *µ*_max_. Yields were calculated by plotting the biomass against glucose concentration and obtaining the slope of the line obtained after linear regression.

## Supplementary information

**Additional file 1.** Additional tables and figures.

**Additional file 2.** Microarray fold changes.

**Additional file 3.** RNA-seq fold changes.

## Data Availability

RNASeq, Lipidomics, Microarray, Excel sheets. Raw transcriptomic data are available in ArrayExpress (accession numbers: E-MTAB-8696, E-MTAB-8716).

## Abbreviations

RB: Regular bubbles
MB: Microbubbles
