# Supplementary material for: A microbubble-sparged yeast propagation–fermentation process for bioethanol production
Source: Biotechnol Biofuels. 2020 Jun 8;13:104. doi: 10.1186/s13068-020-01745-5 (PMC7281951; doi:10.1186/s13068-020-01745-5)
Supplement: Supplementary file 1 — Additional file 1. Additional tables and figures. [file 13068_2020_1745_MOESM1_ESM.docx]

**Additional information**

# Table S1: Differentially regulated genes (≥2-fold, adjusted *p* ≤0.05) during microbubble propagation relative to conventional propagation (t=3 h)

| **Systematic name** | **Standard name** | **Fold change** | **Direction** |
| --- | --- | --- | --- |
| *YHL047C* | *ARN2* | 2.4 | Down |
| *YDR099W* | *BMH2* | 3.6 | Up |
| *YPR124W* | *CTR1* | 3.1 | Down |
| *YLR411W* | *CTR3* | 6.2 | Down |
| *YHR055C* | *CUP1-2* | 3.3 | Up |
| *YKR076W* | *ECM4* | 2.2 | Down |
| *YOL158C* | *ENB1* | 2.4 | Down |
| *YMR058W* | *FET3* | 2.5 | Down |
| *YOR382W* | *FIT2* | 4.6 | Down |

# Table S2: Differentially regulated genes (≥2-fold, adjusted *p* ≤0.05) during microbubble propagation relative to conventional propagation (t=10 h)

| **Systematic name** | **Standard name** | **Fold change** | **Direction** |
| --- | --- | --- | --- |
| *YJL200C* | *ACO2* | 2.2 | Up |
| *YMR303C* | *ADH2* | 2.6 | Up |
| *YMR170C* | *ALD2* | 2.6 | Down |
| *YMR169C* | *ALD3* | 3.8 | Down |
| *YDR441C* | *APT2* | 2.2 | Up |
| *YOL058W* | *ARG1* | 3.1 | Up |
| *YMR062C* | *ARG7* | 2.3 | Up |
| *YDR127W* | *ARO1* | 2.1 | Up |
| *YBR249C* | *ARO4* | 2.1 | Up |
| *YGL202W* | *ARO8* | 2.3 | Up |
| *YLR356W* | *ATG33* | 2.2 | Down |
| *YPL250C* | *ATG41* | 2.0 | Up |
| *YIL140W* | *AXL2* | 2.8 | Up |
| *YAL061W* | *BDH2* | 2.4 | Down |
| *YDR099W* | *BMH2* | 2.7 | Up |
| *YNL269W* | *BSC4* | 2.5 | Down |
| *YPL111W* | *CAR1* | 2.2 | Down |
| *YGR108W* | *CLB1* | 2.1 | Up |
| *YPL256C* | *CLN2* | 3.0 | Up |
| *YDR204W* | *COQ4* | 2.0 | Down |
| *YNL130C* | *CPT1* | 2.1 | Up |
| *YGR189C* | *CRH1* | 2.2 | Up |
| *YOL007C* | *CSI2* | 2.0 | Up |
| *YPR124W* | *CTR1* | 4.7 | Down |
| *YHR055C* | *CUP1-2* | 4.0 | Up |
| *YEL039C* | *CYC7* | 2.2 | Down |
| *YHR019C* | *DED81* | 2.3 | Up |
| *YLR348C* | *DIC1* | 2.3 | Up |
| *YER124C* | *DSE1* | 2.1 | Down |
| *YEL030W* | *ECM10* | 2.2 | Down |
| *YAL003W* | *EFB1* | 2.0 | Up |
| *YHR007C* | *ERG11* | 2.7 | Up |
| *YMR202W* | *ERG2* | 2.2 | Up |
| *YGR060W* | *ERG25* | 2.1 | Up |
| *YLR056W* | *ERG3* | 2.6 | Up |
| *YML008C* | *ERG6* | 2.0 | Up |
| *YGR052W* | *FMP48* | 2.5 | Down |
| *YKR009C* | *FOX2* | 2.0 | Down |
| *YLR214W* | *FRE1* | 2.1 | Down |
| *YCR098C* | *GIT1* | 2.8 | Up |
| *YKL026C* | *GPX1* | 2.2 | Down |
| *YER055C* | *HIS1* | 2.1 | Up |
| *YKL101W* | *HSL1* | 2.2 | Up |
| *YCR021C* | *HSP30* | 2.9 | Down |
| *YPL280W* | *HSP32* | 2.6 | Down |
| *YPL280W* | *HSP32* | 2.6 | Down |
| *YJR016C* | *ILV3* | 2.1 | Up |
| *YEL001C* | *IRC22* | 2.1 | Up |
| *YNL045W* | *LAP2* | 2.1 | Down |
| *YJR010W* | *MET3* | 2.0 | Up |
| *YDL079C* | *MRK1* | 2.3 | Down |
| *YGL236C* | *MTO1* | 2.3 | Up |
| *YNL200C* | *NNR1* | 2.4 | Down |
| *YPL196W* | *OXR1* | 2.0 | Down |
| *YMR174C* | *PAI3* | 2.2 | Down |
| *YOL084W* | *PHM7* | 2.2 | Down |
| *YBR296C* | *PHO89* | 2.8 | Up |
| *YIL160C* | *POT1* | 2.4 | Down |
| *YDR300C* | *PRO1* | 2.2 | Up |
| *YKR013W* | *PRY2* | 2.5 | Up |
| *YJR094W-A* | *RPL43b* | 2.0 | Up |
| *YJL136C* | *RPS21b* | 2.2 | Up |
| *YDR303C* | *RSC3* | 2.1 | Up |
| *YIL113W* | *SDP1* | 2.2 | Down |
| *YAL067C* | *SEO1* | 2.1 | Up |
| *YER096W* | *SHC1* | 2.0 | Down |
| *YMR118C* | *SHH3* | 3.5 | Down |
| *YLR164W* | *SHH4* | 2.9 | Down |
| *YMR175W* | *SIP18* | 3.6 | Down |
| *YMR322C* | *SNO4* | 2.5 | Down |
| *YGR236C* | *SPG1* | 3.7 | Down |
| *YMR107W* | *SPG4* | 3.7 | Down |
| *YPL163C* | *SVS1* | 2.1 | Up |
| *YPR074C* | *TKL1* | 2.0 | Up |
| *YNL300W* | *TOS6* | 2.2 | Up |
| *YGL186C* | *TPN1* | 2.0 | Up |
| *YKR050W* | *TRK2* | 2.0 | Down |
| *YER090W* | *TRP2* | 2.1 | Up |
| *YGR209C* | *TRX2* | 2.1 | Down |
| *YPL207W* | *TYW1* | 2.3 | Up |
| *YMR271C* | *URA10* | 2.1 | Down |
| *YNL283C* | *WSC2* | 2.2 | Up |
| *YLR070C* | *XYL2* | 2.4 | Down |
| *YCL042W* | *YCL042W* | 2.2 | Down |
| *YCL048W-A* | *YCL048W-A* | 4.0 | Down |
| *YDL218W* | *YDL218W* | 2.0 | Down |
| *YDR034W-B* | *YDR034W-B* | 2.7 | Down |
| *YDR222W* | *YDR222W* | 2.2 | Up |
| *YER121W* | *YER121W* | 2.6 | Down |
| *YGR066C* | *YGR066C* | 2.1 | Down |
| *YGR174W-A* | *YGR174W-A* | 2.3 | Down |
| *YGR204C-A* | *YGR204C-A* | 2.4 | Down |
| *YHR020W* | *YHR020W* | 2.2 | Up |
| *YHR210C* | *YHR210C* | 2.1 | Down |
| *YJL144W* | *YJL144W* | 2.8 | Down |
| *YJR115W* | *YJR115W* | 2.7 | Down |
| *YKL065W-A* | *YKL065W-A* | 3.1 | Down |
| *YKL107W* | *YKL107W* | 3.0 | Down |
| *YLR446W* | *YLR446W* | 2.0 | Down |
| *YML083C* | *YML083C* | 2.1 | Down |
| *YNL144C* | *YNL144C* | 2.2 | Down |
| *YNR071C* | *YNR071C* | 2.4 | Down |
| *YOR186W* | *YOR186W* | 3.2 | Down |
| *YPL119C-A* | *YPL119C-A* | 2.1 | Down |

# Table S3: Functional classes enriched in the list of differentially regulated genes during microbubble propagation relative to conventional propagation (t=3 h). Genes with ≥2-fold change and an adjusted *p* of ≤0.05 were used as input in the Funspec online software without Bonferroni correction and with the default *p-*value cutoff.

| **Category** | ***p*-value** | **In Category from Cluster** |
| --- | --- | --- |
| iron ion homeostasis [GO:0055072] | 3.93E-14 | *ARN1 ARN2 FRE6 FRE1 FET3 ENB1 FIT2* |
| ion transport [GO:0006811] | 2.49E-13 | *ARN1 ARN2 FRE6 FRE1 CTR3 FET3 ENB1 FIT2 CTR1* |
| copper ion import [GO:0015677] | 1.20E-09 | *FRE6 FRE1 CTR3 CTR1* |
| siderophore transport [GO:0015891] | 5.28E-07 | *ARN1 ARN2 FIT2* |
| transport [GO:0006810] | 1.31E-06 | *VMR1 ARN1 ARN2 FRE6 FRE1 CTR3 FET3 ENB1 FIT2 CTR1* |
| copper ion transport [GO:0006825] | 2.68E-06 | *FRE1 CTR3 CTR1* |
| copper ion transmembrane transport [GO:0035434] | 1.44E-05 | *CTR3 CTR1* |
| response to copper ion [GO:0046688] | 4.80E-05 | *CUP1-2 FET3* |
| ferric-enterobactin transport [GO:0015685] | 0.00227169 | *ENB1* |
| siderophore metabolic process [GO:0009237] | 0.00227169 | *ARN2* |
| iron assimilation by reduction and transport [GO:0033215] | 0.00453857 | *FET3* |
| detoxification of copper ion [GO:0010273] | 0.00453857 | *CUP1-2* |
| detoxification of cadmium ion [GO:0071585] | 0.00453857 | *CUP1-2* |
| electron transport chain [GO:0022900] | 0.00532629 | *FRE6 FRE1* |
| intracellular sequestering of iron ion [GO:0006880] | 0.00680064 | *FRE6* |
| negative regulation of ubiquitin-protein ligase activity involved in mitotic cell cycle [GO:0051436] | 0.00680064 | *BMH2* |
| removal of superoxide radicals [GO:0019430] | 0.00905791 | *CUP1-2* |
| high-affinity iron ion transport [GO:0006827] | 0.00905791 | *FET3* |

# Table S4: Functional classes that were enriched in the list of differentially regulated genes during microbubble propagation relative to conventional propagation (t=10 h). Genes with ≥2-fold change and an adjusted *p* ≤0.05 was used as input in the Funspec online software without Bonferroni correction and with the default *p-*value cutoff.

| **Category** | ***p*-value** | **Genes in the category** |
| --- | --- | --- |
| cellular amino acid biosynthetic process [GO:0008652] | 0.0000191 | *ARO4 ARO1 PRO1 HIS1 TRP2 MET3 ILV3 ARG7 ARG1* |
| ergosterol biosynthetic process [GO:0006696] | 0.0000226 | *ERG25 ERG11 ERG3 ERG6 ERG2* |
| steroid biosynthetic process [GO:0006694] | 0.0000347 | *ERG25 ERG11 ERG3 ERG6 ERG2* |
| oxidation-reduction process [GO:0055114] | 7.37777E-05 | *BDH2 ARO1 ERG25 ERG11 GPX1 YKL107W FOX2 ERG3 XYL2 FRE1 ALD3 ALD2 ADH2 TYW1* |
| sterol biosynthetic process [GO:0016126] | 7.38816E-05 | *ERG25 ERG11 ERG3 ERG6 ERG2* |
| biological process [GO:0008150] | 8.63065E-05 | *BDH2 YCL042W YCL048W-A YDL218W YDR034W-B YDR222W APT2 IRC22 YER121W YGR066C YGR174W-A YGR204C-A SPG1 YHR210C ACO2 YJR115W YKL065W-A YKL107W PRY2 YLR164W YLR446W YML083C SPG4 YMR118C SNO4 YNL144C YNL200C BSC4 TOS6 YNR071C CSI2 PHM7 YOR186W YPL119C-A HSP32* |
| beta-alanine biosynthetic process [GO:0019483] | 0.000241002 | *ALD3 ALD2* |
| metabolic process [GO:0008152] | 0.000270872 | *BDH2 ARO4 ARO1 CRH1 POT1 ACO2 ILV3 YKL107W FOX2 XYL2 ERG6 ARG7 ALD3 ALD2 ADH2 LAP2 TKL1* |
| polyamine catabolic process [GO:0006598] | 0.000715631 | *ALD3 ALD2* |
| aromatic amino acid family biosynthetic process [GO:0009073] | 0.000732152 | *ARO4 ARO1 TRP2* |
| electron transport chain [GO:0022900] | 0.000926683 | *CYC7 TRX2 YLR164W FRE1 YMR118C* |
| lipid biosynthetic process [GO:0008610] | 0.00121721 | *ERG25 ERG11 ERG3 ERG6 ERG2* |
| hexose metabolic process [GO:0019318] | 0.00141668 | *YHR210C YNR071C* |
| cellular response to water deprivation [GO:0042631] | 0.00141668 | *YJL144W SIP18* |
| copper ion import [GO:0015677] | 0.0063466 | *FRE1 CTR1* |
| G2/M transition of mitotic cell cycle [GO:0000086] | 0.00909881 | *RSC3 CLB1 HSL1* |
| arginine biosynthetic process [GO:0006526] | 0.0099945 | *ARG7 ARG1* |
| fatty acid beta-oxidation [GO:0006635] | 0.0099945 | *POT1 FOX2* |
| response to oxidative stress [GO:0006979] | 0.0099945 | *YDR222W GPX1* |
| phosphate transport [GO:0006817] | 0.0099945 | *PHO89 DIC1* |

# Table S5: Genes that were differentially regulated ≥2-fold (adjusted *p* ≤0.05) during early fermentation using microbubble propagated yeast relative to yeast propagated with regular bubbles (t=7 h)

| **Systematic name** | **Standard name** | **Fold change** | **Direction** |
| --- | --- | --- | --- |
| *YBR085W* | *AAC3* | 2.1 | Down |
| *YNR044W* | *AGA1* | 2.0 | Up |
| *YPL250C* | *ATG41* | 2.9 | Up |
| *YDR099W* | *BMH2* | 4.0 | Up |
| *YPL111W* | *CAR1* | 2.7 | Down |
| *YPR124W* | *CTR1* | 2.7 | Down |
| *YLR411W* | *CTR3* | 3.4 | Down |
| *YGR088W* | *CTT1* | 2.0 | Up |
| *YHR055C* | *CUP1-2* | 4.1 | Up |
| *YCR098C* | *GIT1* | 2.3 | Down |
| *YFL014W* | *HSP12* | 3.6 | Up |
| *YCR021C* | *HSP30* | 4.7 | Up |
| *YML058W-A* | *HUG1* | 2.2 | Down |
| *YOL081W* | *IRA2* | 2.1 | Up |
| *YJL100W* | *LSB6* | 3.9 | Up |
| *YBR195C* | *MSI1* | 2.1 | Up |
| *YPR149W* | *NCE102* | 2.4 | Up |
| *YPR052C* | *NHP6a* | 2.1 | Down |
| *YKR046C* | *PET10* | 2.1 | Up |
| *YDR077W* | *SED1* | 2.4 | Up |
| *YHR008C* | *SOD2* | 2.0 | Up |
| *YGR248W* | *SOL4* | 2.6 | Up |
| *YHR136C* | *SPL2* | 2.5 | Down |
| *YGR048W* | *UFD1* | 2.1 | Down |
| *YBL029C-A* | *YBL029C-A* | 2.0 | Up |
| *YBL071C-B* | *YBL071C-B* | 2.0 | Down |
| *YBR182C-A* | *YBR182C-A* | 2.4 | Up |
| *YCR061W* | *YCR061W* | 2.4 | Up |
| *YER053C-A* | *YER053C-A* | 4.9 | Up |
| *YGR234W* | *YHB1* | 2.0 | Up |
| *YHR007C-A* | *YHR007C-A* | 3.4 | Up |
| *YIL156W-B* | *YIL156W-B* | 2.0 | Down |
| *YKL126W* | *YPK1* | 2.3 | Down |
| *YPR078C* | *YPR078C* | 2.0 | Down |

# Table S6: Functional classes enriched in the list of differentially regulated genes during early fermentation for microbubble propagated yeast relative to yeast propagated with regular bubbles (t=7 h).

| **Category** | ***p*-value** | **Genes in the category** |
| --- | --- | --- |
| plasma membrane organization [GO:0007009] | 0.0000257 | *HSP12 NCE102* |
| copper ion transmembrane transport [GO:0035434] | 0.0000770 | *CTR3 CTR1* |
| cell adhesion [GO:0007155] | 0.0005318 | *HSP12 AGA1* |
| eisosome assembly [GO:0070941] | 0.0005318 | *YPK1 NCE102* |
| copper ion import [GO:0015677] | 0.0007068 | *CTR3 CTR1* |
| response to stress [GO:0006950] | 0.0009821 | *HSP30 HSP12 CTT1 YHB1 IRA2* |
| Ras protein signal transduction [GO:0007265] | 0.0019374 | *MSI1 BMH2* |
| copper ion transport [GO:0006825] | 0.0019374 | *CTR3 CTR1* |
| response to temperature stimulus [GO:0009266] | 0.0051492 | *SPL2* |
| cellular response to osmotic stress [GO:0071470] | 0.0051492 | *HSP12* |
| negative regulation of phospholipid translocation [GO:0061093] | 0.0051492 | *YPK1* |
| arginine catabolic process to ornithine [GO:0019547] | 0.0051492 | *CAR1* |
| regulation of ornithine metabolic process [GO:0090368] | 0.0051492 | *CAR1* |
| negative regulation of protein phosphorylation [GO:0001933] | 0.0051492 | *NCE102* |
| glycerophosphodiester transport [GO:0001407] | 0.0051492 | *GIT1* |

# Table S7: Genes that were differentially regulated ≥2-fold (adjusted *p* ≤0.05) during late fermentation using microbubble propagated yeast relative to yeast propagated with regular bubbles (t=32 h)

| **Systematic name** | **Standard name** | **Fold change** | **Direction** |
| --- | --- | --- | --- |
| YML054C | CYB2 | 3.3 | Up |

# Table S8: Functional classes enriched in the list of differentially regulated genes during early microbubble gassed fermentation relative to fermentation with regular bubbles (t=4 h) using yeast cells that were propagated using microbubbles. Genes with ≥2-fold change in expression and an adjusted *p* of ≤0.05 were used as input in the Funspec online software without Bonferroni correction and with the default *p-*value cutoff.

| Category | p-value | In Category from Cluster |
| --- | --- | --- |
| response to stress [GO:0006950] | 3.37E-11 | SSA1 FRT2 TIP1 TPS1 SSE2 TPS2 HSP42 AHA1 HSP78 TIR1 SSA4 HSP12 MDJ1 JAC1 HSF1 CTT1 YHB1 GRE3 YVH1 KAR2 HSP150 SSC1 YKL069W SSA2 HSP104 UBI4 IRC20 HSP60 TSL1 HSC82 HOR7 YDJ1 OCA1 FMP41 IRA2 YGK3 STI1 HSP82 ATH1 |
| protein refolding [GO:0042026] | 2.64E-09 | SSA1 SSE2 HSP78 MDJ1 SSC1 CPR6 HSP60 HSC82 YDJ1 HSP10 HSP82 |
| ribosome biogenesis [GO:0042254] | 5.07E-08 | MAK5 ENP1 REI1 TSR1 NOP6 FAL1 UTP5 RRP17 UTP6 UTP7 CGR1 ROK1 SDA1 PXR1 RPL8A SSF1 IPI1 DBP8 RIX1 FAF1 HCA4 ALB1 DHR2 DBP7 UTP30 RLP24 ESF2 NOP8 UTP23 BUD21 RIO1 PNO1 YTM1 RRS1 NIP7 DIM1 |
| mitochondrial electron transport, ubiquinol to cytochrome c [GO:0006122] | 2.31E-07 | QCR7 RIP1 CYC7 QCR6 QCR9 QCR8 CYC1 CYT1 |
| protein folding [GO:0006457] | 1.48E-06 | SSA1 CNS1 SSE2 PDI1 CPR1 AHA1 SSA4 MDJ1 JAC1 SSC1 SBA1 SSA2 CPR6 HSP60 FPR4 TSA1 CIN4 HSC82 SIS1 YDJ1 HSP10 STI1 HSP82 |
| electron transport chain [GO:0022900] | 4.04E-06 | COR1 GRX2 QCR7 RIP1 CYC7 QCR6 QCR9 QCR8 CYC1 SDH1 SDH2 CYB2 FRE7 CYT1 FRE5 |
| rRNA processing [GO:0006364] | 4.81E-06 | POP8 MAK5 ENP1 TSR1 NOP6 FAL1 RRP1 UTP5 RRP17 UTP6 SNM1 UTP7 CGR1 ROK1 POP6 EFG1 PXR1 RPP1 IPI1 DBP8 RIX1 HCA4 DHR2 DBP7 UTP30 FYV7 TSR2 RNT1 ESF2 REX4 UTP23 BUD21 YTM1 NIP7 DIM1 |
| cellular response to oxidative stress [GO:0034599] | 1.55E-05 | NTG1 PRX1 UGA2 YDL124W GRX2 HSP12 ACT1 GRE3 URM1 YKL069W LTV1 MCR1 LOT6 AHP1 TSA1 GAD1 OCA1 |
| 'de novo' protein folding [GO:0006458] | 1.90E-05 | MDJ1 HSP60 HSC82 YDJ1 HSP82 |
| proteasomal ubiquitin-dependent protein catabolic process [GO:0043161] | 2.34E-05 | PRE7 SHP1 PRE1 RPN11 PRE4 VID30 VID28 FYV10 PRE8 PRE6 PRE2 |
| ubiquitin-dependent protein catabolic process [GO:0006511] | 2.36E-05 | RPT2 RPN6 CDC48 RPN5 UBX5 RPN3 DDI1 UFD1 RPN1 RPN2 RPT1 UBP11 PRE8 UBP15 PRE6 RPT5 RPN7 |
| oxidation-reduction process [GO:0055114] | 2.76E-05 | PRX1 UGA2 YDL124W SFA1 GLT1 HEM13 DIT2 RIP1 DLD3 CTT1 YHB1 AIM17 GRE3 COX5B KGD1 GUT2 AAD10 MAE1 YKL069W SDH1 MCR1 URA1 MTD1 SDH2 LOT6 AHP1 TSA1 CYB2 DUS1 NDI1 GCV2 IDH1 ZWF1 GOR1 FRE7 IDH2 DFR1 ALD4 FRE5 FDH1 IRC15 ALD6 |
| tRNA methylation [GO:0030488] | 5.89E-05 | TRM8 TRM1 TRM5 GCD14 TRM112 TRM11 TRM13 PPM2 |
| proteasome assembly [GO:0043248] | 7.88E-05 | RPN6 BLM10 PRE4 ECM29 RPN2 HSC82 HSP82 |
| response to heat [GO:0009408] | 0.0002063 | MDJ1 HSF1 PIL1 YAP1 YDJ1 SGT2 LSP1 |
| metabolic process [GO:0008152] | 0.0002551 | NTG1 NTH2 UGA2 YBR056W SFA1 GLT1 TPS2 APA2 URA3 HPA3 PHM8 CEM1 GSY1 PMC1 PNC1 PYC1 SER2 IMA1 FUR1 DCD1 KGD1 RPE1 INO1 FBP26 CPA2 BAT2 MAE1 UBA1 URA1 MTD1 PDC5 DPH5 ACO1 YLR345W CYB2 NTE1 OGG1 DUS1 YMR084W YMR090W ADE17 ERG12 ADE4 IDH1 FMP41 ZWF1 GOR1 IDH2 FAA1 ALD4 FDH1 ULA1 ALD6 ATH1 GDB1 |
| glutamate biosynthetic process [GO:0006537] | 0.0002851 | CIT2 GLT1 ACO1 IDH1 CIT1 IDH2 |
| proteolysis involved in cellular protein catabolic process [GO:0051603] | 0.0003143 | PRE7 PRE1 PRE4 PRE8 PRE6 PEP4 PRE2 |
| purine nucleotide biosynthetic process [GO:0006164] | 0.0003143 | ADE1 MTD1 ADE13 ADE17 ADE4 ADE12 ADE2 |
| 'de novo' IMP biosynthetic process [GO:0006189] | 0.0003252 | ADE1 ADE13 ADE17 ADE4 ADE2 |
| proteasomal ubiquitin-independent protein catabolic process [GO:0010499] | 0.000465 | PRE7 PRE1 PRE4 PRE8 PRE6 PRE2 |
| citrate metabolic process [GO:0006101] | 0.0005262 | CIT2 ACO1 CIT1 |
| protein unfolding [GO:0043335] | 0.0005262 | HSP78 SSC1 HSP104 |
| trehalose biosynthetic process [GO:0005992] | 0.001215 | TPS1 TPS2 TSL1 PGM2 |
| NADPH regeneration [GO:0006740] | 0.001215 | PYC1 ZWF1 ALD4 ALD6 |
| tRNA processing [GO:0008033] | 0.001626 | POP8 TRM8 TRM1 POP6 RPP1 TRM5 URM1 GCD14 TRM2 DUS1 MSS1 TRM11 TRM13 PPM2 MOD5 |
| glycogen biosynthetic process [GO:0005978] | 0.001665 | GLC3 GSY1 GLG1 PGM2 GDB1 |
| vacuolar protein catabolic process [GO:0007039] | 0.001665 | APE3 PRB1 VID30 VID28 PEP4 |
| glycine metabolic process [GO:0006544] | 0.001978 | GCV1 SHM2 GCV2 |
| regulation of catalytic activity [GO:0050790] | 0.001978 | RPN3 RPN1 RPN2 |
| glucose 6-phosphate metabolic process [GO:0051156] | 0.001978 | GLK1 EMI2 PGM2 |
| fructose 2,6-bisphosphate metabolic process [GO:0006003] | 0.001978 | PFK26 FBP26 YLR345W |
| base-excision repair, AP site formation [GO:0006285] | 0.001978 | NTG1 MAG1 OGG1 |
| positive regulation of protein catabolic process [GO:0045732] | 0.001978 | RPT2 RPN4 RPT1 |
| tricarboxylic acid cycle [GO:0006099] | 0.002056 | CIT2 KGD1 SDH1 SDH2 ACO1 IDH1 CIT1 IDH2 |
| ribosomal large subunit biogenesis [GO:0042273] | 0.002203 | MAK5 REI1 SDA1 ALB1 RLP24 NOP8 YTM1 RRS1 NIP7 |
| ribosomal large subunit assembly [GO:0000027] | 0.002685 | LSG1 SSF1 IPI1 RIX1 YVH1 DBP7 RPF2 REX4 NIP7 |
| protein catabolic process [GO:0030163] | 0.002961 | RPT2 BLM10 RPT1 RPT5 RPT4 SUE1 |
| endonucleolytic cleavage in ITS1 to separate SSU-rRNA from 5.8S rRNA and LSU-rRNA from tricistronic rRNA transcript (SSU-rRNA, 5.8S rRNA, LSU-rRNA) [GO:0000447] | 0.003895 | ENP1 UTP6 UTP7 DBP8 ESF2 UTP23 BUD21 PNO1 RRS1 |
| regulation of protein catabolic process [GO:0042176] | 0.004647 | RPN3 RPN1 RPN2 |
| peptidyl-diphthamide biosynthetic process from peptidyl-histidine [GO:0017183] | 0.004647 | KTI11 DPH2 DPH5 |
| tRNA modification [GO:0006400] | 0.005147 | TRM8 TRM2 DUS1 MSS1 MOD5 |
| one-carbon metabolic process [GO:0006730] | 0.005147 | GCV1 MTD1 SHM2 GCV2 DFR1 |
| proteasome regulatory particle assembly [GO:0070682] | 0.005986 | RPT2 RPT1 RPT5 RPT4 |
| arginine biosynthetic process [GO:0006526] | 0.005986 | ARG4 ARG3 CPA2 ARG1 |
| SRP-dependent cotranslational protein targeting to membrane, translocation [GO:0006616] | 0.005986 | SSA1 SSA4 KAR2 SSA2 |
| mitochondrial citrate transport [GO:0006843] | 0.006529 | CTP1 YHM2 |
| negative regulation of nucleotide metabolic process [GO:0045980] | 0.006529 | STF1 INH1 |
| ceramide catabolic process [GO:0046514] | 0.006529 | YPC1 YDC1 |
| ceramide metabolic process [GO:0006672] | 0.006529 | YPC1 YDC1 |
| negative regulation of cAMP biosynthetic process [GO:0030818] | 0.006529 | IRA1 IRA2 |
| plasma membrane organization [GO:0007009] | 0.006529 | HSP12 NCE102 |
| positive regulation of telomere maintenance via telomerase [GO:0032212] | 0.006529 | SBA1 HSP82 |
| endonucleolytic cleavage to generate mature 5'-end of SSU-rRNA from (SSU-rRNA, 5.8S rRNA, LSU-rRNA) [GO:0000472] | 0.007056 | UTP6 UTP7 DBP8 ESF2 UTP23 BUD21 PNO1 |
| maturation of SSU-rRNA from tricistronic rRNA transcript (SSU-rRNA, 5.8S rRNA, LSU-rRNA) [GO:0000462] | 0.00782 | FAL1 UTP5 UTP6 UTP7 EFG1 FAF1 DHR2 FYV7 TSR2 BUD21 RIO1 |
| carbohydrate metabolic process [GO:0005975] | 0.007969 | GAL7 YBR056W GLK1 EMI2 GLC3 IMA1 YHR210C RPE1 YMR085W PGM2 ZWF1 YNR071C GAS4 ATH1 GDB1 |
| fructose metabolic process [GO:0006000] | 0.008739 | PFK26 FBP26 YLR345W |
| protein import into mitochondrial intermembrane space [GO:0045041] | 0.008739 | HOT13 MIA40 HSP60 |
| negative regulation of catalytic activity [GO:0043086] | 0.008739 | STF1 INH1 PRB1 |
| pyrimidine nucleotide biosynthetic process [GO:0006221] | 0.008812 | URA3 DCD1 URA1 URA4 |

# Table S9: Functional classes enriched in the list of differentially regulated genes during early microbubble gassed fermentation relative to fermentation with regular bubbles (t=12 h). Genes with ≥2-fold change in expression and an adjusted *p* of ≤0.05 were used as input in the Funspec online software without Bonferroni correction and with the default *p-*value cutoff.

| Category | *p*-value | In Category from Cluster |
| --- | --- | --- |
| heme catabolic process [GO:0042167] | 0.003029 | *HMX1* |
| heme oxidation [GO:0006788] | 0.003029 | *HMX1* |
| neutral amino acid transport [GO:0015804] | 0.003029 | *PUT4* |
| sterol biosynthetic process [GO:0016126] | 0.00337 | *PDR16 HES1* |
| proline transport [GO:0015824] | 0.006049 | *PUT4* |
| response to zinc ion [GO:0010043] | 0.006049 | *IZH1* |
| positive regulation of translational termination [GO:0045905] | 0.006049 | *ANB1* |
| anaerobic respiration [GO:0009061] | 0.006049 | *AAC3* |
| amino acid transport [GO:0006865] | 0.00698 | *BTN2 PUT4* |
| positive regulation of translational elongation [GO:0045901] | 0.009061 | *ANB1* |
| proline catabolic process [GO:0006562] | 0.009061 | *PUT4* |
| gamma-aminobutyric acid transport [GO:0015812] | 0.009061 | *PUT4* |
| lactate metabolic process [GO:0006089] | 0.009061 | *CYB2* |
| cell cycle arrest [GO:0007050] | 0.009061 | *HUG1* |
| electron transport chain [GO:0022900] | 0.009413 | *CYC1 CYB2* |

# Table S10: Functional classes enriched in the list of differentially regulated genes during early microbubble gassed fermentation relative to fermentation with regular bubbles (t=44 h). Genes with ≥2-fold change in expression and an adjusted *p* of ≤0.05 were used as input in the Funspec online software without Bonferroni correction and with the default *p-*value cutoff.

| Category | *p*-value | In Category from Cluster |
| --- | --- | --- |
| disaccharide catabolic process [GO:0046352] | 0.0004768 | *IMA1 IMA2* |
| maltose metabolic process [GO:0000023] | 0.001717 | *IMA1 IMA2* |
| protection from non-homologous end joining at telomere [GO:0031848] | 0.005755 | *RAP1* |
| GMP salvage [GO:0032263] | 0.005755 | *HPT1* |
| IMP salvage [GO:0032264] | 0.005755 | *HPT1* |
| azole transport [GO:0045117] | 0.005755 | *AZR1* |
| amine transport [GO:0015837] | 0.005755 | *DTR1* |
| positive regulation of sister chromatid cohesion [GO:0045876] | 0.005755 | *SPO13* |
| meiosis [GO:0007126] | 0.007015 | *RDH54 SPO23 SPO13 REC102* |
| cellular bud site selection [GO:0000282] | 0.009608 | *BUD4 BUD17* |

# Table S11: Differentially expressed genes during microbubble fermentation (t=12 h) relative to fermentation with regular bubbles were ranked according to documented DNA binding and expression evidence by the indicated transcription factor to identify enriched regulons.

| **Transcription factor** | | **% in user set^a^** | **% in**  ***S. cerevisiae^b^*** | ***p-*value** | **Target genes** |
| --- | --- | --- | --- | --- | --- |
| Hap1p | 41.67 | | 1.65 | 0.000001379262422 | *IZH1 LSB6 ANB1 CYC1 HMX1 CYB2 MOT3 NDE1 PDR16 PUT4* |

^a^Percentage of differentially expressed gene that are targets for the transcription factor

^b^Percentage of transcription factor target genes relative to the whole genome

**Figures**

:

# Figure S1: Yeast cell budding index during the fed-batch fermentation. Yeast cultures were propagated aerobically, either in the prototype microbubble fermenter or in an unmodified Infors bioreactor, and sparged with 21% oxygen as described in the main text. Propagation took place for 10 h, followed by 45 h of fermentation. Circles (regular bubbles) and squares (microbubbles). The data are the means and standard deviation (n=4).

# Figure S2: Levels of free amino nitrogen during the fed-batch fermentation. Yeast cultures were propagated aerobically, either in the prototype microbubble fermenter or in an unmodified Infors bioreactor, and sparged with 21% oxygen as described in the main text. Propagation took place for 10 h, followed by 45 h of fermentation. Circles (regular bubbles); squares (microbubbles). Free amino nitrogen was assayed as described in .ASBC method of analysis (<http://methods.asbcnet.org/summaries/wort-12.aspx>). The data are the means and standard deviation (n=4).

# **Figure S3:** Yeast cell budding index during gassed (with 1% oxygen) fed-batch fermentation. Yeast cultures were propagated aerobically in the prototype microbubble fermenter and sparged with 21% oxygen as described in the text. Propagation took place for 10 h, followed by 45 h of fermentation. Circles (regular bubbles) and squares (micro-bubbles). The data are the means and standard deviation (n=3).

# Figure S4 (a) Ethanol concentrations during regular bubble propagated and regular bubble gassed fermentations plotted against those values obtained from microbubble propagated and microbubble gassed fermentations with the prototype microbubble reactor. (b) Ethanol concentrations during regular bubble propagated and ungassed fermentations plotted against those values obtained from microbubble propagated and ungassed fermentation using the prototype microbubble reactor. The data are the mean and standard deviations (n=3 for Figure 4a and n = 4 for Figure 4b)

# Figure S5: Yeast cell viability during nitrogen gassed fermentations employing microbubble propagated using 21% oxygen as inocula. Circles (regular bubbles) and squares (microbubbles). The data are the means and standard deviation (n=2).
